# Coronavirus (COVID-19) pandemic mediated changing trends in nuclear medicine education and training: time to change and scintillate

**DOI:** 10.1007/s00259-021-05241-2

**Published:** 2021-03-04

**Authors:** Gopinath Gnanasegaran, Diana Paez, Mike Sathekge, Francesco Giammarile, Stefano Fanti, Arturo Chiti, Henry Bom, Sobhan Vinjamuri, Thomas NB Pascual, Jamshed Bomanji

**Affiliations:** 1 Institute of Nuclear Medicine, University College London Hospital, Tower 5, 235 Euston Road, London, NW1 2BU UK; 2 Royal Free London NHS Foundation Trust Hospital, London, UK; 3 Division of Human Health, International Atomic Energy Agency, Vienna, Austria; 4 Nuclear Medicine Department, University of Pretoria and Steve Biko Academic Hospital, Pretoria, South Africa; 5 Department of Experimental, Diagnostic and Specialty Medicine, University of Bologna, Bologna, Italy; 6 Humanitas University and Humanitas Research Centre, Milan, Italy; 7 Department of Nuclear Medicine, Chonnam National University, Seoul, South Korea; 8 Royal Liverpool University Hospital, Liverpool, L7 8XP UK; 9 Philippine Nuclear Research Institute, Department of Science and Technology, Quezon City, Philippines

## Introduction

The world is amidst a COVID-19 pandemic. It started in December 2019, when a new type of coronavirus 2019-nCoV/SARS-CoV-2, causing the COVID-19 disease, was extracted and identified from the patient’s lower respiratory tract samples in Wuhan, China [1]. The pandemic has shaken the social, economic, political, environmental, and technology worldwide. Specifically, it shook the scientific and health structures worldwide, and we are still yet to recover as new strains of the virus pose a further threat to society. When writing this article, the World Health Organization (WHO) has confirmed cases 101,406,059 of COVID-19, including 2,191,898 deaths [2]. There is a significant strain on health care systems to provide essential services amidst the pandemic while protecting the health care workers and patients. Several countries are currently offering the COVID-19 vaccine to people at high risk and several frontline workers. The World Health Organization (WHO) published COVID-19: Operational Guidance for maintaining essential health services during an outbreak [3]. In the nuclear medicine community, the International Atomic Energy Agency (IAEA) organized a series of webinars on the coronavirus disease (COVID-19) pandemic as it affects nuclear medicine services and discussed the challenges including education and training [4, 5].

Globally, educational institutions, students of all ages, and their parents face tremendous stress and dilemma about the future as schools and universities have closed, and assessments are postponed or cancelled. In the medical world, various national and international bodies aim to develop alternative pathways to deal with these situations for immediate implementation and the future. However, the task is challenging. The current COVID-19 pandemic poses many challenges for the practice of nuclear medicine and has changed the way we work and train future nuclear medicine physicians [4–6]. There will be several changes that might take place in how we would like to teach our future nuclear medicine physicians. Therefore, a coordinated response from the global nuclear medicine community is paramount to achieve this task. The nuclear medicine community and its policymakers should be ready for the change to meet the new demands of education and training. We should develop innovative and effective teaching methodologies to provide optimal training for future nuclear medicine physicians while utilizing many available online educational resources within the community. In this editorial, we discuss the challenges for teaching nuclear medicine trainee physicians and provide options and opportunities to support teaching and training to prepare for the new normal.

## Nuclear medicine education and training challenges

The pandemic affected the personal and professional lives of health care workers globally. In addition to maintaining the smooth functioning of clinical services, health care institutions must provide adequate uninterrupted post-graduate nuclear medicine physician training. A proportion of trainees might have been re-deployed to work in the clinical wards and intensive care units [7–10]. Staff isolation, physical distancing, remote working, and limited-service provision leading to reduced case volume and range for reporting have further complicated the situation. In 2020, radionuclide therapy was stopped or postponed in most countries subject to regional pandemic incursions. In most of the cases, the cancellation of mandatory clinical rotation for junior trainees (residents) and cancellation of assessment methods such as fellowship exams for senior trainees (residents) added to the stress and welfare of the trainees [7–10]. For international students, travel restrictions might pose a significant problem for attending taught courses or clinical fellowships. Research programmes and their students cannot progress or complete their projects as most projects are delayed or stopped due to the pandemic. There is limited scope for starting new research projects or enrolment for higher research degrees.

In the initial phase of the pandemics, the departments’ focus was mainly to clear the backlog. These gave a limited option for teaching. In the later stage, the challenges included a limited number of staff in the reporting rooms, with requirements for physical distancing leading to minimal or no in-person supervision to report scans at the physical workstation or to supervise procedures (injections or performing therapies). There was limited presential teaching, including face-to-face interaction between supervisors and trainees or mentorship [7–11]. The regular face-to-face teaching sessions and journal clubs have suffered and, in some cases, have come to a standstill and/or are morphing into online sessions. Given the intermittent lockdowns and periodic spikes in the COVID-19 cases, this will take time to return to normality as we know it [7–11]. Essentially, in a short span, nuclear medicine physicians’ teaching and training are now shifting from traditional structured face-to-face methods to emergency remote teaching to the more flexible blended-learning approach being adopted in the new normal [12, 13].

## Available resources and solutions

Providing regular teaching and training for professionals is often challenging. The onus is not only on local departments but also the responsibility of national and international societies. The following sections briefly describe how different providers can contribute to nuclear medicine physicians’ training in pandemic and beyond through suggested educational and organizational strategies and the utility of available resources.

## Nuclear medicine departments

In general, conventional nuclear medicine training is delivered through scan reporting sessions and didactic lectures and taught programmes in several countries with exit fellowship exams. With the current pandemic, face-to-face teaching and training sessions are limited by law. Therefore, we are moving from a face-to-face learning atmosphere to a complete virtual digital environment. Preparation for emergency remote teaching is required in addition to online education [12–14]. In general, emergency remote teaching is a temporary measure. The main objective is to provide rapid access to instruction quickly and reliably during a crisis. However, this should not re-design or re-create existing robust educational system, specifically the curriculum [12–14]. We can transition education and training from live face-to-face teaching (through case-based discussion, journal clubs, etc.) to online meeting platforms. This can be further enhanced by playing pre-recorded lectures or webinars, followed by live discussion and self-assessment questions. These can be delivered to individual trainees or groups of trainees/residents from the same hospital or different hospitals worldwide. For example, the diagnostic and radionuclide therapy procedures performed per the department’s local rules should be recorded and viewed by the staff on demand without interrupting the clinical services, followed by self-assessment. In nuclear medicine, most case strength is image-based. The training departments will need to develop a local database of different scan type short videos of all the departments’ procedures, including technical aspects (e.g. performing daily QC/QA, radiolabelling methods). The database will help in self-learning and self-assessment of trainees with more flexibility.

**Fig. 1:**
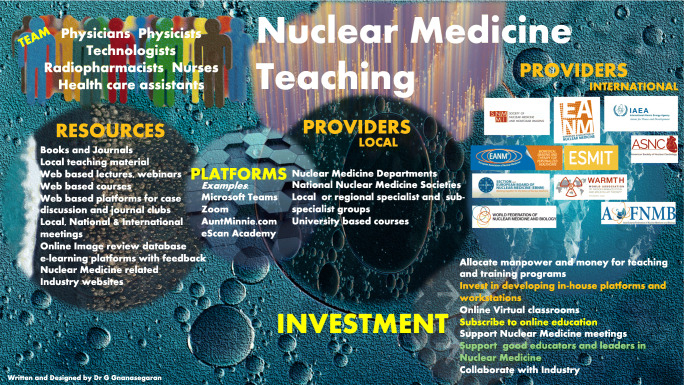
Nuclear medicine educational resources, platforms, providers, and future investment (kindly note: The authors are not promoting the platforms or organizations shown in this figure or mentioned in this article. The websites, apps, methodology, technology quality, and content are ever-changing. The authors advise all readers to review the aforementioned websites, societies, and material for content and quality before using or recommending them. The views and opinions expressed in this article are those of the authors and do not necessarily reflect the official policy or position of any other agency, organization, association, employer, or company)

**Fig. 2:**
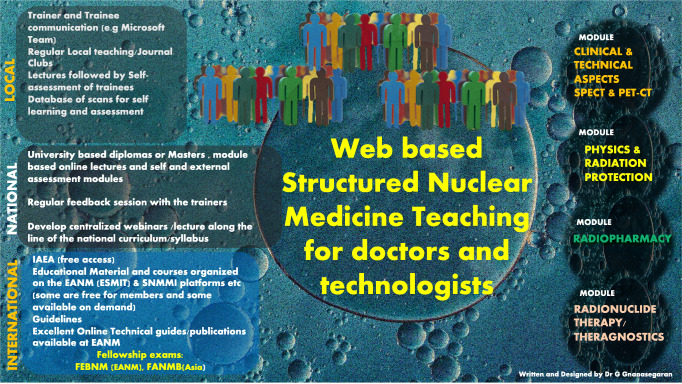
Web-based structured nuclear medicine teaching and training for doctors and technologists

In non-training centres and smaller nuclear medicine departments, the most straightforward way to provide continuous education is to play or view the free access or member access on-demand lectures offered online. In the new normal scenario, it is expected that most meetings will probably become virtual or a combination of face-to-face and online (Figs. 1 and 2). The most cost-effective way of providing regular updates is by departments making some provisions for registering for online access meetings, which will benefit the trainees and staff. However, all these ideas require sincere passion, commitment from the local team, and funding from the institution.

## National nuclear medicine training bodies/committees

Nuclear medicine training’s current mission should be focused on cooperative learning strategies and building a community of nuclear medicine physician learners interested in sharing their ideas to study nuclear medicine based on the prescribed national curriculum [15, 16]. In general, they should allocate some funding to the training centres to develop educational modules related to the national training curriculum for nuclear medicine. The more straightforward and cost-effective way of supporting this venture would be to identify training centres that specialize in a particular procedure or identify a specific faculty member interested in their choice to provide specific modules. For example, a faculty or department renowned for radionuclide cardiac imaging can develop webinars related to performing the procedure and interpreting scans [15]. This model will (a) prevent duplication of education material; (b) avoid wasting resources, workforce, and funding; (c) standardize training; and (d) promote equal accessibility and opportunity for trainees nationally. For this approach to reach its full potential, the nuclear medicine faculties and departments should be generous in sharing their experience and resources. Although we may work at different institutions, our joint aim should be towards better patient care. Therefore, we should share if we care.

## National nuclear medicine societies

The contribution of national nuclear medicine societies to training and teaching is variable. This heterogeneity could be related to several factors, such as the organization’s size, budget, expectation, and commitment. Most societies assume their role in supporting teaching and training by organizing an annual conference. This only partially fulfils some learning and teaching objectives towards continuous medical education (CME) and clinical professional development (CPD). Nuclear medicine societies should organize regular topic-based online discussions or lectures on current and future trends to educate and update their members, including trainees and trainers. The simplest way for societies to support their members is to select high-quality lectures from previous annual meetings and create an online teaching resource for its members after the presenter’s permission. To deliver this does not require additional funding resources and is the most cost-effective method. The American Society of Nuclear Cardiology’s classic example produced extremely valuable webinars and documents during the lockdown. Their willingness to promote education training should be appreciated and welcomed, and this example should be followed by societies interested in facilitating training and teaching.

## International nuclear medicine societies

Several dynamic international societies such as the European Association of Nuclear Medicine (EANM) and the Society of Nuclear Medicine and Molecular Imaging (SNMMI) sincerely promote the specialty by providing high-quality meetings practicing nuclear medicine professionals and trainees (Table 1). They also organize regular sub-specialty based or topic-based symposiums.

**Table 1:** International nuclear medicine related societies and some of their educational activities, resources, and platforms (kindly note: The authors are not promoting the platforms or organizations shown in this figure or mentioned in this article. The websites, apps, methodology, technology quality, and content are ever-changing. The authors advise all readers to review the aforementioned websites, societies, and material for content and quality before using or recommending them. The views and opinions expressed in this article are those of the authors and do not necessarily reflect the official policy or position of any other agency, organization, association, employer, or company)

Organization and websites	Educational materials	Courses and certification
European Association of Nuclear Medicine (EANM) www.eanm.org	1. European Nuclear Medicine Guide—free access 2. Technologists guides—Free access 3. ESMIT webinars on PET/CT, basic nuclear medicine on the ESMIT e-learning platform 4. Guidelines—free access 5. *European Journal of Nuclear Medicine and Molecular Imaging* (EJNMMI) 6. EJNMMI Physics 7. EJNMMI Research 8. *European Journal of* Hybrid Imaging	1. The European School of Multimodality Imaging & Therapy (ESMIT) courses 2. Radiopharmacy certification 3. Focus meetings 4. Certificate of fellowship of the European Board of Nuclear Medicine
Society of Nuclear Medicine & Molecular Imaging (SNMMI) www.snmmi.org	1. Guidelines—free access, online lectures 2. *The Journal of Nuclear Medicine* (JNM) 3. *Journal of Nuclear Medicine* 4. Technology (JNMT) audio lectures with PowerPoint 5. Teaching cases with slides (non-credit) 6. Study guides 7. Textbooks with online assessment	1. Online education programmes, short, journal-based courses to timely online lectures, to comprehensive review courses 2. Review courses and workshops for physicians, technologists, physicists, and pharmacists
International Atomic Energy Agency (IAEA) www.iaea.org humanhealth.iaea.org	1. DATOL (online training resources for nuclear medicine professionals) 2. Nuclear medicine and PET-CT (teaching cases, lectures) 3. Webinars 4. E-learning modules 5. Guidelines and IAEA publications 6. NUMDAB (NUclear Medicine DAtaBase) 7. Quality Management Audits in Nuclear Medicine Practice (QUANUM)	Regular IAEA training courses
American Society of Nuclear Cardiology (ASNC) Primary source for education in the field of nuclear cardiology www.asnc.org ASNC learning centre	1. Guidelines 2. *Journal of Nuclear Cardiology* (JNC) 3. Online CME and webinars (e.g. ASNC virtual CV molecular imaging seminars) 4. Best practices in nuclear cardiology: a webinar series 5. Board certification resources 6. Nuclear cardiology knowledge self-assessment modules	1. Board exam prep course 2. Nuclear cardiology board exam 3. ASNC nuclear cardiology virtual elective 4. Online nuclear cardiology training curriculum: open access
Asian Regional Cooperative Council for Nuclear Medicine (ARCCNM) www.arccnm.org www.aofnmb.org	e-learning modules http://www.rcaro.org/elearning/	Certificate exam: Fellow of the Asian Nuclear Medicine Board (FANMB)

The EANM has developed the European School of Multimodality Imaging & Therapy (ESMIT), dedicated to teaching and training and represents EANM’s response to massive changes in the nuclear medicine community’s educational needs. They cater to different groups. Until recently, these were delivered as partly online webinars and partly face-to-face. However, we hope most of the material will be available on the online platform at some stage. The fee for this programme is reported to be nominal. Similar high-quality educational lectures and programmes are also public within the SNMMI platform. However, access to all these educational materials comes with a cost and is often subsidized for its members. The International Atomic Energy Agency (IAEA) provides excellent teaching resources on its platform, covering the entire spectrum of nuclear medicine in its open-access website: IAEA Human Health Campus [17, 18]. The IAEA faculty work hard in producing high-quality educational materials, which are excellent and popular in developing countries. The Regional Cooperative Agreement Regional Office (RCARO) provides an e-learning platform for nuclear medicine. The Asian Regional Cooperative Council for Nuclear Medicine (ARCCNM) faculties and fellows of the Asian Nuclear Medicine Board (ANMB) have worked together to produce e-learning modules on nuclear medicine’s essential knowledge for physicians. Currently, it provides 50 learning modules for free access.

The British Institute of Radiology (BIR) in the UK has developed PET-CT webinar series, which has 15–20 cancer-based lectures and is open access. Clinicians and trainees from all over the world can access these lectures by just registering on the website and will receive CPD points at the end of the session. Shortly, you will access a series of webinars on radionuclide therapy (theragnostics) topics from the BIR. The American Society of Nuclear Cardiology (ASNC), a primary source for education in nuclear cardiology, provides an excellent platform to physicians and technologists. Each issue of the *Journal of Nuclear Cardiology* now features an article designated for continuing education credit for physicians and technologists.

The World Association of Radiopharmaceutical and Molecular Therapy (WARMTH) is making commendable efforts to promote radionuclide therapy worldwide. They organize extraordinary meetings towards the science and practice of radionuclide therapies. Under the WARMTH umbrella, the World Theragnostic Academy (WTA) is developing a radionuclide training platform, which will be functional soon. The WTA platform will provide a great opportunity and accessibility to training and teaching radionuclide therapies. Besides, there is good collaboration between nuclear medicine associations with organizations such as the European Society for Radiotherapy and Oncology (ESTRO) and the European School of Oncology (ESO). We wish to express our sincere gratitude to all these organizations and their members for their selfless contribution to education.

## Industry

The industries supporting both nuclear medicine imaging and therapies play an important role in providing valuable training and teaching to their consumers. They are pro-active in promoting and supporting teaching and training, which is encouraging. Currently, most vendors regularly organize online teaching/webinars to support education and training, which should be welcomed and appreciated. However, the industry should also work closely with national societies or individual departments to develop online/web-based teaching and training platforms.

## Independent educational platforms

There are several excellent independent educational platforms such as Radiopaedia, Aunt Minnie, and eScan Academy. The passion, selfless commitment, hard work, and dedication of the volunteers are highly commendable. Aunt Minnie is another excellent resource with a dedicated nuclear medicine section, which is extremely useful. eScan Academy is an online education resource for nuclear medicine specialists, residents, and students. It contains valuable educational material that can be viewed via individual or institutional paid access. In particular, the institutional access option may be useful for training centres if it removes the need for them to develop their platform, which is likely to be time-consuming and will require funding and immense selfless dedication from the developers.

## Advantages and challenges of digital learning

**Fig. 3:**
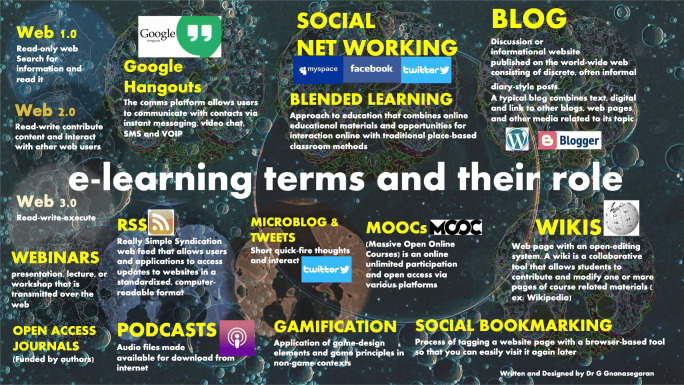
Some e-learning terms and their applications (adapted from ref 19) (kindly note: The authors are not promoting the platforms or organizations shown in this figure or mentioned in this article. The websites, apps, methodology, technology quality, and content are ever-changing. The authors advise all readers to review the aforementioned websites, societies, and material for content and quality before using or recommending them. The views and opinions expressed in this article are those of the authors and do not necessarily reflect the official policy or position of any other agency, organization, association, employer, or company)

The internet provides phenomenal accessibility to education material and developing them. There are several methods for the online delivery of educational material. Individuals seeking to get involved, whether as an educator/trainer or as a learner/trainee, should have a working knowledge of terms (Fig. 3) [19, 20]. The provider should understand the application capabilities before using them [19]. However, like any other teaching or training methods, it has several advantages and limitations for both the trainers and the trainee (Table 2) [19, 21–28]. The threat of social and intellectual isolation might be secondary to reduced face-to-face interaction in online teaching and training (online learning, e-learning, virtual learning) [19, 21–28]. However, the strength of e-learning or web-based learning is its level playing structure, where everybody gets access to a structural material irrespective of geography or background. Course content, quality, and the “user-friendliness” of delivery platforms play a significant role in delivering useful material [19, 21–28]. However, internet access, connectivity, and hardware might be limiting factors in low-income countries. These should be taken into consideration while developing platforms to provide easy access to downloadable course materials. In general, every organization (and the individual working within them) would want to create educational content and platforms that are considered excellent and may be tempted to replicate or duplicate those that already exist. The requirements for success will be met only when we focus on the nuclear medicine community’s needs as a whole rather than on self-gain. We believe that blended teaching and training, which combines face-to-face and online delivery methods, will be most popular and useful [18–28]. Major limitation still includes limited universal internet access due to technological limitation and physical infrastructure constraints [19–28].

**Table 2:** Advantages and current challenges of e-learning and webinars (19–28)

Advantages	Challenges
**e-Learning**	
1. Help in focussing the needs of individual trainee or faculty members 2. Flexible (time, place, and delivery) 3. Access to a large amount of information 4. Provides learning opportunities for unlimited audience 5. Space and venue are not required or mandatory 6. Eliminates additional expense (e.g. travel, accommodation, food) 7. Improved quality of education 8. Most often affordable 9. Accessibility 10. Lectures can be viewed multiple times 11. Lectures can be updated regularly 12. Reach-specific target group (e.g. nuclear cardiology, paediatric nuclear medicine) 13. Engage and cater specific target group	1. Absence or limited personal interactions 2. Social and emotional distancing 3. Quality of nuclear medicine material is in-homogenous 4. In-homogeneous skill of presenters and providers 5. Assessments might possibly be done with the use of proxy; may pose challenges to regulate activities such as cheating. 6. Not all nuclear medicine topics can be delivered remotely/online (e.g. radionuclide therapies, radiolabelling experience, operating scanners) 7. Good internet access with sufficient bandwidth is essential 8. Not all resources are open access 9. Trainees or delegates might multi-task during the lectures (e.g. texting, emailing, chatting) 10. Not everyone is “tech savvy” 11. Unexpected technical problems while viewing 12. Limited networking opportunities 13. Limited access to practical hands-on training
**Webinars**	
1. Unlimited audience reach or registration 2. Increases the reach and the impact of the information 3. Relatively inexpensive 4. Easy to produce (if experienced) 5. No special equipment required (technology is evolving) 6. Can be recorded in advance so that people can view at their convenience (e.g. according to their schedule or from different time zones) 7. Can be viewed multiple times (on-demand) 8. People from different time zones can view the recorded version at their convenience 9. Cost-effective for both the presenter and the audience in most cases. 10. Affordable platforms are increasing and evolving 11. Reaches specific target group 12. Engages specific target group	1. One-way presentation (with or without an interactive session at the end) 2. Unexpected technical problems during broad casting 3. Audience may get easily distracted 4. Business firewalls could delay access 5. Variable internet speed may result in time lag or interruptions during webinars 6. Variable system configurations might be incompatible 7. Technical difficulties and limited familiarity for the faculty to develop these 8. Lack of control over the audience environment

## Conclusion

COVID-19 puts a strain on the previously structured teaching and learning of nuclear medicine trainees. Efforts are being made to deliver the goals of the training programme amidst the global pandemic. Innovative teaching and learning methods coupled with available online educational resources provide elements that contribute to this goal.

Technological innovations are playing a significant role in our imaging world. It is time for us to keep abreast of change and adapt to digital learning programmes and platforms. These are bound to create online discussion and virtual communities of nuclear medicine physicians, technologists, physicists, radiopharmacists, and nurses.

Although there is good material available online, these resources are of variable quality, and there is a lack of integration between them. The nuclear medicine community should use the existing platforms provided by international nuclear medicine societies rather than reinventing the wheel by developing local platforms. Sincere efforts should be made to popularize these resources to fill the wide gap in nuclear medicine teaching and training globally. Finally, it is time to think seriously about investing in the future of our scintillating specialty, both locally and internationally. We should work together to create simple, workable solutions for safe teaching and learning.
