# Correction to: Coronavirus (COVID-19) pandemic mediated changing trends in nuclear medicine education and training: time to change and scintillate

**DOI:** 10.1007/s00259-022-05966-8

**Published:** 2022-09-23

**Authors:** Gopinath Gnanasegaran, Diana Paez, Mike Sathekge, Francesco Giammarile, Stefano Fanti, Arturo Chiti, Henry Bom, Sobhan Vinjamuri, Thomas NB Pascual, Jamshed Bomanji

**Affiliations:** 1 Institute of Nuclear Medicine, University College London Hospital, Tower 5, 235 Euston Road, London, NW1 2BU UK; 2 Royal Free London NHS Foundation Trust Hospital, London, UK; 3 Division of Human Health, International Atomic Energy Agency, Vienna, Austria; 4 Nuclear Medicine Department, University of Pretoria and Steve Biko Academic Hospital, Pretoria, South Africa; 5 Department of Experimental, Diagnostic and Specialty Medicine, University of Bologna, Bologna, Italy; 6 Humanitas University and Humanitas Research Centre, Milan, Italy; 7 Department of Nuclear Medicine, Chonnam National University, Seoul, South Korea; 8 Royal Liverpool University Hospital, Liverpool, L7 8XP UK; 9 Philippine Nuclear Research Institute, Department of Science and Technology, Quezon City, Philippines

**Correction to: European Journal of Nuclear Medicine and Molecular Imaging (2022) 49:427–435**

**https://doi.org/10.1007/s00259-021-05241-2**

The article Coronavirus (COVID-19) pandemic mediated changing trends in nuclear medicine education and training: time to change and scintillate, written by Gopinath Gnanasegaran, Diana Paez, Mike Sathekge, Francesco Giammarile, Stefano Fanti, Arturo Chiti, Henry Bom, Sobhan Vinjamuri, Thomas NB Pascual, and Jamshed Bomanji, was originally published online on March 4, 2021 with Open Access under a Creative Commons Attribution 4.0 International License. After publication in volume 49, issue 2, page 427–435 the author(s) decided to cancel the Open Access. Therefore, the copyright of the article has been changed on September 6, 2022 to © The Author(s), under exclusive licence to Springer-Verlag GmbH Germany, part of Springer Nature 2022 with all rights reserved.

